# Synchronous Occurrence of Splenic Pleomorphic Mantle Cell Lymphoma and Esophageal Adenocarcinoma with Overexpression of BCL1 Protein

**DOI:** 10.1155/2020/8888829

**Published:** 2020-12-21

**Authors:** Dominik Dabrowski, Roberto F. Silva, Michael Constantinescu, Rodney E. Shackelford, Nestor Dela Cruz, Eric X. Wei

**Affiliations:** ^1^Department of Pathology and Translational Pathobiology, Louisiana State University Health Sciences Center Shreveport, Shreveport, LA 71130, USA; ^2^Department of Pathology and Microbiology, University of Nebraska, Omaha, NE 68198, USA; ^3^Department of Pathology, Overton Brooks Veterans Affairs Medical Center, Shreveport, LA 71101, USA

## Abstract

Synchronous occurrences of mantle cell lymphoma (MCL), or intermediate lymphocytic lymphoma, and other malignancies are rare. Such cases present diagnostic and especially therapeutic challenges, making them of particular interest to study. We report a case of synchronic MCL and an esophageal tumor in an elderly male patient. Morphologically, the tumors were classified as splenic pleomorphic MCL and adenocarcinoma of the esophagus occurring concurrently. The pleomorphic MCL mimicked diffuse large B cell lymphoma (DLBCL) but lacked larger centroblast- or immunoblast-like cells. Curiously, both tumors overexpressed cyclin D1 by immunohistochemistry. This is an important feature that distinguishes MCL pathologically from two of its closest entities in the differential diagnosis: chronic lymphocytic leukemia and DLBCL, the latter of which mantle cells cannot transform into. The lymphoproliferation revealed IGH/CCND1 translocation by FISH, but the esophageal adenocarcinoma only showed CCND1 aneuploidy without break-apart signals. Since the gastrointestinal (GI) tract is a common site of extranodal involvement by MCL and lymphomatous polyposis can present as GI polyps, adequate care was taken to differentiate the esophageal adenocarcinoma from advanced stagings of MCL, as well as metastatic adenocarcinoma. Despite numerous immunohistochemical stainings studied, only BCL1 was demonstrated to have partial overlap in both tumors. The patient underwent esophagectomy and splenectomy. A subsequent metastatic primary lung squamous cell carcinoma was diagnosed, after which the patient expired. MCL typically presents at an advanced stage and has been deemed incurable with a prognosis of only several years. It is unclear whether the patient succumbed to complications of his MCL or the metastatic squamous cell carcinoma. Furthermore, he was lost to follow-up for a year and only received treatment after his third cancer was diagnosed. We have reviewed previous reports of synchronic mantle cell lymphoma and other solid tumors or hematological malignancies in the literature.

## 1. Introduction

Mantle cell lymphoma (MCL) is a mature B cell lymphoproliferative disorder that is found in about 3–10% of all adult non-Hodgkin's lymphomas (NHL) in the United States [1]. The neoplasm is composed of small- to medium-sized lymphocytes with irregular nuclear contours, and more than 95% of these tumors reveal a CCND1 gene translocation. According to Chandran et al., the incidence of MCL has recently increased [2]. This disorder is aggressive, incurable neoplasia frequently diagnosed at an advanced stage in Caucasian males between the sixth and eighth decades of life. MCL usually requires treatment due to its rapid growth, and its clinical outcome has significantly improved recently. However, in contrast to other aggressive lymphomas, it often relapses after several years of remission [3]. Increased long-term risks of developing secondary neoplasms in the thyroid, esophagus, and stomach have been described in the NHL survivors [4], but a histopathological diagnosis of 2 synchronous malignancies in a patient with any lymphoma is exceedingly rare. In this report, we present a case of an elderly male patient diagnosed with an MCL pleomorphic subtype, an infrequent variant of the disorder, and an esophageal adenocarcinoma, both with overexpression of BCL1. This last feature raised concerns about the underlying clonal and molecular pathophysiology of both neoplasms and its clinical significance for the patient. It is of interest that this patient was later found to also be diagnosed with stage IV poorly differentiated squamous cell carcinoma of the lung and subsequently expired, suggesting his overall genomic instability and propensity to malignancy. The patient was subsequently treated at a federal hospital for his third and final cancer. Extensive regulatory restrictions prohibited further tissue procurement and advanced studies of his squamous cell carcinoma. The patient was considered to have both synchronous, defined as occurring within two months of one another, and metachronous, defined as greater than six months apart, tumors [5]. For reasons already described, we choose to focus on the two earlier synchronous cancers of this patient.

## 2. Case Report

A 71-year-old type II diabetic, smoker, and hypertensive Caucasian male patient presented to the surgical service of our hospital for evaluation of splenomegaly and pancytopenia. A laboratory workup found that he had anemia (hematoglobin 7.0 g/dL), leukopenia (white blood count 2.19 k/*μ*L), thrombopenia (platelets 50 k/*μ*L), and hypoalbuminemia (3.1 g/dL). He complained of upper left abdominal pain but denied fever, chills, nausea, or vomiting. A physical exam revealed no lymphadenopathy, but his left upper quadrant of the abdomen was tender on palpation, and splenomegaly was observed. An upper gastrointestinal endoscopy showed an esophageal polyp, which by pathological examination revealed Barrett's esophagus with high-grade dysplasia and evolving invasive adenocarcinoma. Due to his symptomatology, and the concern of the possible spread of either cancer, he underwent transhiatal esophagectomy, splenectomy, and cholecystectomy. He was found to have splenic pleomorphic MCL and esophageal adenocarcinoma by pathological examination. His MCL international prognostic index, which consists of age, performance status, LDH level, and leukocyte count, was 7 out of 11 points, placing him in the high-risk category. In this category, his median survival, from the time of diagnosis, would be predicted to be less than 3 years [6]. This would later turn out to be accurate. The patient did not have a bone marrow biopsy for lymphoma staging. Thus, his karyotype status was unavailable. The patient did not go through further chemotherapy in our oncology clinic after the operation. Follow-up for over 6 months by the surgical oncology was focused on the treatment of his postsurgical esophageal stenosis. The patient was subsequently lost to follow-up, but one year later, at another hospital, he presented with recurrent bouts of aspiration pneumonia. Radiology and bronchoscopy examinations revealed a right lower lobe lung mass. The endobronchial biopsy revealed soft tan tissues at 0.7 × 0.6 × 0.2 cm in aggregate, which turned to be poorly differentiated squamous cell carcinoma of the lung. Staging positron emission tomography (PET) scan found the lung mass at 9 cm in the greatest dimension and the tumor metastasis to the right clavicle, lower trapezius muscle, and regional lymph nodes. It was determined he had stage IV pulmonary squamous cell carcinoma. 10 days after one cycle of chemotherapy, he was hospitalized for incontinence and severe back pain. Magnetic resonance imaging (MRI) showed spinal lytic lesions at the levels of T8 and L4. During the hospitalization, he developed streptococcal bacteremia, which resulted in his port-a-cath being removed. Despite the second round of chemotherapy after discharge, the patient expired shortly thereafter.

**Figure 1:**
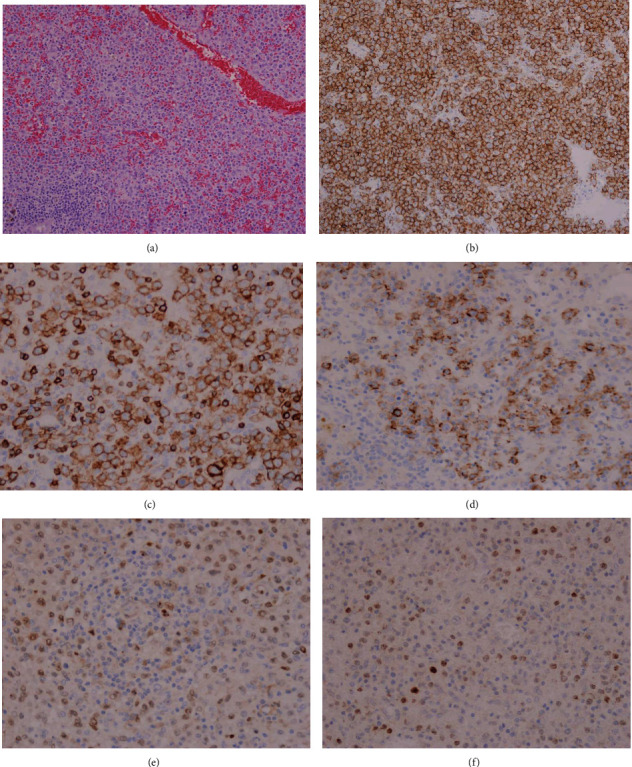
Pleomorphic mantle cell lymphoma involves the spleen. (a) Diffuse infiltration of the splenic red pulp by large-sized tumor cells with clumped chromatin and conspicuous nucleolus (100x). (b) Immunostain for CD20 demonstrates membranous pattern positivity (200x). (c) Immunostain for CD5 reveals membranous pattern positivity (400x). (d) Immunostain for CD23 exhibits partial membranous pattern positivity (400x). (e) Immunostain for cyclin D1 demonstrates partial nuclear positivity (400x). (f) Immunostain for SOX11 presents with partial nuclear pattern positivity (400x).

**Figure 2:**
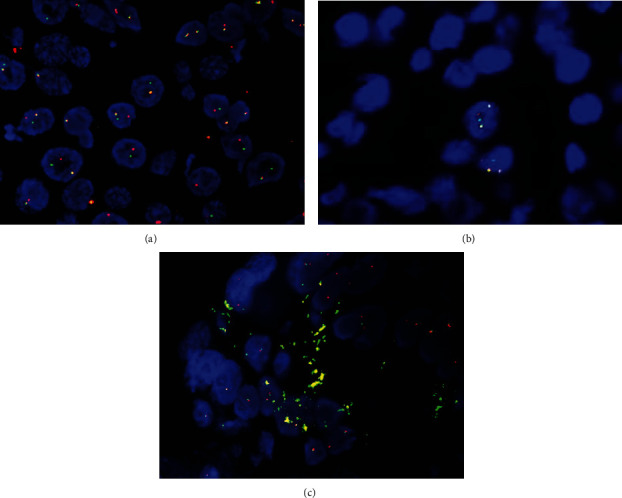
BCL1 FISH for splenic pleomorphic mantle cell lymphoma and esophageal adenocarcinoma. (a) The splenic mantle cell lymphoma shows red and green break-apart signals in 41% of cells by LSI BCL1 dual-color FISH probes (400x). (b) The lymphoma reveals BCL1/IGH dual-fusion signals in 65% of tumor cell nuclei with CCND1 and IGH gene copy numbers at 2 to 4 copies per cell (400x). (c) Esophageal adenocarcinoma exhibits no BCL1/CCND1 gene rearrangement in all the interphase tumor cells analyzed. However, aneuploidy with the single gene copy number of BCL1/CCND1 is observed in 24% of cells scored (400x).

**Figure 3:**
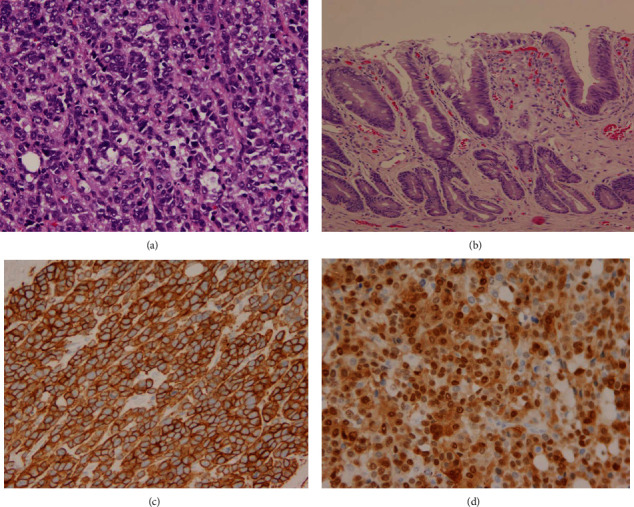
Esophageal adenocarcinoma arises from Barrett's esophagus. (a) Poorly differentiated invasive adenocarcinoma involving the submucosa of the esophagus (200x). (b) The adenocarcinoma originates from overlying Barrett's esophagus with high-grade dysplasia (200x). (c) Immunostain for AE1/AE3 demonstrates strong cytoplasmic and membrane pattern positivity (200x). (d) Esophagus, immunostain for cyclin D1 demonstrates strong nuclear pattern positivity (200x).

The gross examination of his splenectomy specimen revealed a massive spleen that weighed 2639 grams and measured 30 × 19 × 9 cm. The splenic parenchyma exhibited homogeneous congested cut surfaces without overt nodularity. A splenic hilar lymph node at 1.3 cm in diameter was also dissected. A 4.2 × 3.8 × 0.4 cm partially circumferential, granular, tan-red ulcerated mass was found in the lower third of the esophagus which proximally displaced the Z-line. Some small lymph nodes in the esophageal adventitia and gastric serosa were identified. The gallbladder was morphologically normal. The microscopic study of the spleen displayed a diffuse involvement of the red pulp by a large lymphoid cell population with irregular nuclear contours and conspicuous nucleoli (Figure 1(a)). The splenic hilar lymph node demonstrated a total effacement of the architecture by the neoplastic lymphoid cells with a similar appearance to those in the red pulp. Ancillary testing performed on sections from the splenic tumor cells demonstrated strong immunoreactivity for CD20 (Figure 1(b)), PAX5, CD5 (Figure 1(c)), and BCL2; partial reactivity for CD23 (Figure 1(d)), CD45, BCL1 (Figure 1(e)), and SOX11 (Figure 1(f)); and negativity for AE1/AE3, BCL6, CD10, c-MYC, MUM1, p53, and S-100. The tumor cell proliferation index was at 30-40% by Ki-67 staining. The splenic lymphoma revealed BCL1/CCND1 break-apart signals in 41% tumor cells, using a locus-specific probe (LSI BCL1 Dual Color Break Apart, Abbott Molecular, Abbott Park, IL) for BCL1/CCND1 on 11q13 (Figure 2(a)). BCL1/IGH translocation fusion signals were detected in 65% tumor nuclei using probes for the CCND1 gene at 11q13 and the immunoglobulin heavy chain gene at 14q32 (Integrated Oncology, Phoenix, AZ) (Figure 2(b)). Both the CCND1 and IGH genes showed two to four copy numbers in lymphoma cells. The esophagus was affected by a poorly differentiated adenocarcinoma infiltrating into the submucosal layer (Figure 3(a)). The adenocarcinoma arose from Barrett's esophagus with high-grade dysplasia (Figure 3(b)). Fourteen lymph nodes in the periesophageal and perigastric connective tissue were negative for metastatic carcinoma or mantle cell lymphoma. The pathologic stage for the esophageal adenocarcinoma was pT1b, pN0, which correlates to a stage 1B cancer. The esophageal adenocarcinoma exhibited strong staining for AE1/AE3 (Figure 3(c)), BCL1 (Figure 3(d)), and p53 and was negative for synaptophysin and CD56. The FISH for the BCL1/CCND1 gene performed on the esophageal tumor showed aneuploidy in 24% of the tumor cells scored (Figure 2(c)), without gene rearrangement or break-apart signals for the CCND1 gene. Microscopic examination of his subsequent endobronchial lung biopsy one and a half years later revealed poorly differentiated squamous cell carcinoma predominantly in sheeting patterns that had pleomorphic nuclei and dense cytoplasm, with occasional keratinized cells noted. Some of the tumor cells were clustered in an acinar fashion. Immunohistochemical staining showed the lung tumor cells were positive for BER-EP4, CK5/6, CK7, CK19, and p63 and negative for CD56, CK20, chromogranin, Napsin A, synaptophysin, and TTF-1. Despite extensive communication with the hospital that had diagnosed and treated the patient's pulmonary squamous cell carcinoma, the patient's pathological imaging and additional tissue materials could not be obtained due to federal restrictions. For this reason, our discussion focuses almost exclusively on the synchronous MCL and esophageal adenocarcinoma as described.

## 3. Discussion

We presented a challenging case with synchronous splenic pleomorphic mantle cell lymphoma and esophageal adenocarcinoma and subsequently metachronous pulmonary squamous cell carcinoma. The patient had the classical demographic features of mantle cell lymphoma but did not present with B symptoms. Splenomegaly is a frequent feature in these patients. Anemia, leukopenia, thrombopenia, and hypoalbuminemia are often present. These features are frequently described in patients with the advanced stage of diffuse large B cell lymphoma (DLBCL) and a pleomorphic variant of MCL [7].

The first histopathologic impression of the spleen was DLBCL. A weak pattern of immunoreactivity for CD23 has been observed in a scant number of cases of MCL [1, 7]. SOX11, a very sensitive marker of MCL, is highly variable in staining reactivity between laboratories. It was mildly positive in the tumor cells in our case, along with BCL1 immunohistochemistry, but this feature is essential in the distinction of a pleomorphic variant of MCL from DLBCL [8]. The pleomorphic MCL is rare and occurs in only 1–3% of MCL [7, 8]. The negativity for c-MYC, BCL6, and CD10 was also in concordance with the diagnosis of MCL. It has been reported that blastoid and pleomorphic variants may show more frequent c-MYC staining than a typical MCL [9, 10]. The cytogenetic hallmark of MCL is t(11;14) or IGH/CCND1 translocation that places the CCND1 gene under the control of the IGH locus enhancer element. Cytogenetic studies are more comprehensive than FISH and molecular testing, but break-apart FISH can detect rare CCND1 translocations and much smaller chromosomal abnormalities. As a result of the translocation, cyclin D1 is overexpressed in MCL lymphocytes exerting an oncogenic activity. Its association with cyclin-dependent kinase 4/6 (CDK 4/6) promotes inactivation of the retinoblastoma protein 1 (RB1) by phosphorylation, inducing cell cycle progression from the G1-to-S phase [11].

A key diagnostic feature in our diagnosis of the MCL was the FISH positivity of BCL1-CCND1 (cyclin D1) break-apart and BCL1/IGH translocation. However, one of the most interesting findings in this case was the overexpression of BCL1 and p53 on the esophageal adenocarcinoma. In a breast cancer study of 90 patients, Bukholm et al. found overexpression of cyclin D1 in 42.2% of the tumors and 10 cases showed amplification of the cyclin D1 locus [12]. No relationship was found between p53 and cyclin D1 status in those cases, but wild-type p53 and lower grade tumors were more frequent in cyclin D1-positive lesions. In gastric and gastroesophageal junction adenocarcinomas, Bar-Sela et al. found 89.5% of the cases stained positive or highly positive for cyclin D1, but there was no correlation between the level of expression of the protein and clinicopathological parameters or survival [13]. Lehrbach et al. also found expression of p53 in 68% and cyclin D1 in 18.7% of 75 cases of esophagogastric junction adenocarcinomas [14].

There have been reports of synchronic MCL and CML [15], MCL and laryngeal neuroendocrine carcinoma [16], MCL and adenocarcinoma or squamous carcinoma of the lung [17–21], which incidentally now includes our case, MCL and genitourinary tract tumors [22–25], MCL and breast cancer [26, 27], MCL associated with the gastrointestinal tract and endocrine tumors [4, 28–31], and MCL and papillary thyroid carcinoma [4]. An esophageal adenocarcinoma associated with MCL has not been described in any of these series; neither has an overexpression of cyclin D1 in both lymphoma cells and carcinoma cells been described. The development of a third cancer in the form of a metastatic squamous cell carcinoma of the lung makes this case all the more unique, indicating patient overall genomic instability and malignant inclination.

## Data Availability

The underlying data supporting the results of the study can be found in the manuscript.
